# Stem Cell Mediation of Functional Recovery after Stroke in the Rat

**DOI:** 10.1371/journal.pone.0012779

**Published:** 2010-09-22

**Authors:** Pedro Ramos-Cabrer, Carles Justicia, Dirk Wiedermann, Mathias Hoehn

**Affiliations:** In-vivo-NMR Laboratory, Max Planck Institute for Neurological Research, Cologne, Germany; Julius-Maximilians-Universität Würzburg, Germany

## Abstract

**Background:**

Regenerative strategies of stem cell grafting have been demonstrated to be effective in animal models of stroke. In those studies, the effectiveness of stem cells promoting functional recovery was assessed by behavioral testing. These behavioral studies do, however, not provide access to the understanding of the mechanisms underlying the observed functional outcome improvement.

**Methodology/Principal Findings:**

In order to address the underlying mechanisms of stem cell mediated functional improvement, this functional improvement after stroke in the rat was investigated for six months after stroke by use of fMRI, somatosensory evoked potentials by electrophysiology, and sensorimotor behavior testing. Stem cells were grafted ipsilateral to the ischemic lesion. Rigorous exclusion of spontaneous recovery as confounding factor permitted to observe graft-related functional improvement beginning after 7 weeks and continuously increasing during the 6-month observation period. The major findings were i) functional improvement causally related to the stem cells grafting; ii) tissue replacement can be excluded as dominant factor for stem cell mediated functional improvement; iii) functional improvement occurs by exclusive restitution of the function in the original representation field, without clear contributions from reorganization processes, and iv) stem cells were not detectable any longer after six months.

**Conclusions/Significance:**

A delayed functional improvement due to stem cell implantation has been documented by electrophysiology, fMRI and behavioral testing. This functional improvement occurred without cells acting as a tissue replacement for the necrotic tissue after the ischemic event. Combination of disappearance of grafted cells after six months on histological sections with persistent functional recovery was interpreted as paracrine effects by the grafted stem cells being the dominant mechanism of cell activity underlying the observed functional restitution of the original activation sites. Future studies will have to investigate whether the stem cell mediated improvement reactivates the original representation target field by using original connectivity pathways or by generating/activating new ones for the stimulus.

## Introduction

Stroke is a leading cause of mortality and the main cause of morbidity in developed countries. Thrombolysis is the only effective treatment to restore blood flow and preserve brain function, accepted in the clinic. However, this treatment presents serious safety-related restrictions so that its use is limited to a very small fraction of all stroke patients (less than 3%) [1].

On the other hand, the brain has the potential to spontaneously resolve stroke-related functional deficits. Although the exact mechanisms are not known, among the discussed possibilities are activation of alternative, already existing neuronal networks, rewiring of new circuits, plastic reorganizations, or also promoting tissue repair by endogenous stem cells. Unfortunately, these aspects are not understood and are often not sufficient to compensate the severe damage inflicted by the stroke. With the recent fast progress of stem cell biology, high expectations are set in the use of growth factors and stem cell-based therapies [2] to promote functional recovery after brain damage. Regenerative therapies have, indeed, been demonstrated to be effective in several experimental studies of animal models of stroke [3]–[9].

Most of these published studies have assessed the effectiveness of stem cells promoting functional recovery by behavioral testing. Despite the undoubted contribution of these investigations, the behavioral studies do not provide access to the understanding of the mechanisms underlying the observed functional outcome improvement. For a causally based therapy optimization the potentially contributing mechanisms, i.e. integration of implanted cells and network formation or support of endogenous affected neuronal tissue by paracrine activity, must be recognized and discriminated. Furthermore, it is of high importance to understand the mechanism by which the therapeutic effect of the stem cell activity acts: i.e. recovery enhancement, induction of plastic reorganization or even transhemispheric compensation are all options, explaining a functional improvement as observed by behavior patterns.

Then, the following two major questions arise: i) do the stem cells execute their beneficial effect by tissue integration or rather by paracrine effects; and ii) which mechanism, activated by the cells' therapeutic activity, explains the functional improvement: recovery of the original representation field or plastic reorganization? To address these two questions, we have decided to investigate stem cell mediated functional improvement by use of a non-invasive imaging technique, in particular functional Magnetic Resonance Imaging (fMRI), which allows to detect, localize and characterize activated cortical representation fields to a specific sensorimotor stimulus. Additionally, this strategy has the immense advantage to follow both, lesion evolution and functional deficit, followed by later functional improvement, in individual subjects in a longitudinal manner, rather than to rely on cross-sectional studies with multiple subjects at different time points.

Taking these considerations into account, we have designed and performed a longitudinal study of stem cell mediated functional recovery after stroke, using a well-established experimental model of ischemic stroke, together with a protocol for longitudinal functional assessment of individual animals by combined use of fMRI, somatosensory evoked potentials (SSEPs) and sensorimotor behavioral testing, over a period of 6 months after ischemic lesion induction and stem cell implantation [10]–[11].

The same experimental protocol was used in the past to characterize spontaneous functional recovery after stroke [10]. This fact helped us in the present investigation to separate spontaneous functional improvements from those achieved only by the presence of stem cells in the cerebral parenchyma.

Our data will show that, when proper conditions for cell-based therapy success are fulfilled, it is possible to claim that stem cells do effectively promote functional recovery after stroke.

## Results

### Protocol of Animal Selection for the Treatment Study

**Figure 1:**
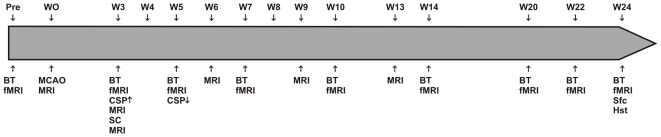
Time schedule of the experimental procedures. BT: behavioral testing, CSP: Cyclosporin application, fMRI: functional MRI study, Hst: histology, MCAO: middle cerebral artery occlusion, i.e. stroke induction, MRI: 3D high-resolution T2*-weighted MRI, SC: stem cell implantation, Sfc: sacrifice, W: week.

**Figure 2:**
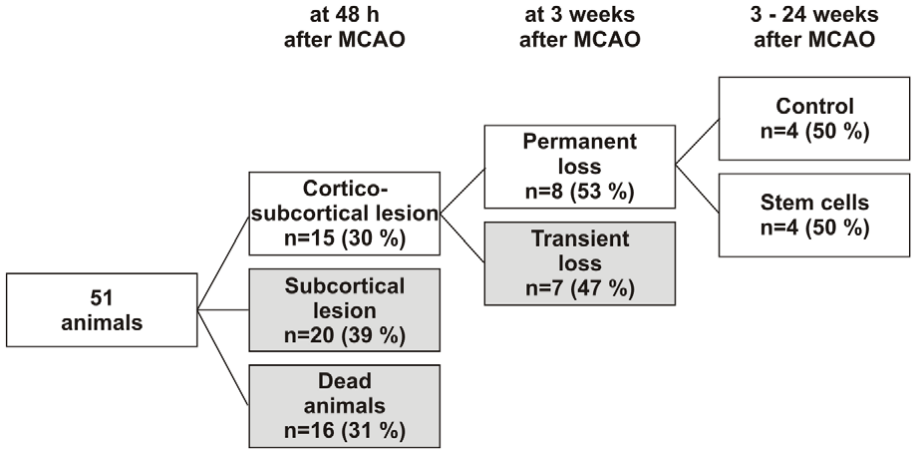
Selection process of the animals for the treatment inclusion. Animals were selected according to the outcome from the MCAO surgery (only subcortical vs. cortico-subcortical lesions) and the fMRI study performed at week 3 (regained vs. non-regained BOLD activation). Finally the remaining animals were randomly asigned to a control and a stem cell-treated group. Gray boxes indicate animals excluded from further procedures at each time point, while white boxes indicate the animals selected for successive stages of the study.

A total of 51 animals entered the longitudinal study, depicted in Fig. 1, and were submitted to middle cerebral artery occlusion (MCAO). Sixteen of them died either during surgery or during the first 48 h, most probably as a consequence of massive edema with swelling of the affected hemisphere and compression of the unaffected hemisphere. Among the survivors (n = 35) from this acute phase, the extension of the ischemic lesion was studied at 48 hours after MCAO by calculating T2 maps from the spin-echo MR images. In a previous fMRI study using the same experimental model, we had already shown that lesions, restricted to the caudate-putamen of the brain and not affecting cortical areas, resulted in animals with no loss or just a transient loss of functional brain activity. This activity was then spontaneously recovered 2–3 weeks after MCAO for all subjects with only striatal lesions [10]. Accordingly, to avoid confounding effects from spontaneous recovery processes on stem cell treated animals, those animals with an exclusively striatal lesion but intact cortex on T2 maps, were excluded from our study at this point, 48 h after stroke induction (20 animals excluded, 39% of the total n = 51 and 57% of the 35 survivors) (see Fig. 2).

The remaining 15 animals with cortico-striatal lesions underwent an fMRI study 18–20 days later (i.e. 3 weeks after MCAO). The period of 3 weeks before the fMRI was established again in accordance with the previously reported results of the study of spontaneous functional recovery following stroke [10]. There, it was stated that 3 weeks after stroke induction can be considered the earliest, safe time point to discriminate animals with spontaneous recovery of brain activity from those with permanent loss of functional activity (defined as absence of any BOLD signal in fMRI studies and as absence of electrical activity registered on somatosensory evoked potentials over the S1_FL_ areas of the cortex) [10].

Therefore, and according to our earlier established discrimination criteria, only those animals without fMRI over the ischemic hemisphere at 3 weeks after MCAO were included for therapeutic intervention of stem cells, thus safely excluding confounding interferences from naturally-occurring spontaneous processes. Seven of the 15 animals assessed at week 3 (47% of the group of 15, 14% of the total n = 51) had regained electrical activity and a BOLD signal and were therefore excluded from the stem cell implantation study.

The remaining 8 animals (53% of the 15 included ones, and 17% of the total n = 51 animals at the beginning of the study) were unambiguously suitable for therapeutic studies. The whole sorting process of the animals from the beginning of the study up to this point is presented in Fig. 2.

### Stem Cell Implantation

The group of n = 8 animals, suitable for the study, was randomly split into two groups of n = 4 animals, group I to be treated with stem cells, and group II to be used as untreated control stroke group.

**Figure 3:**
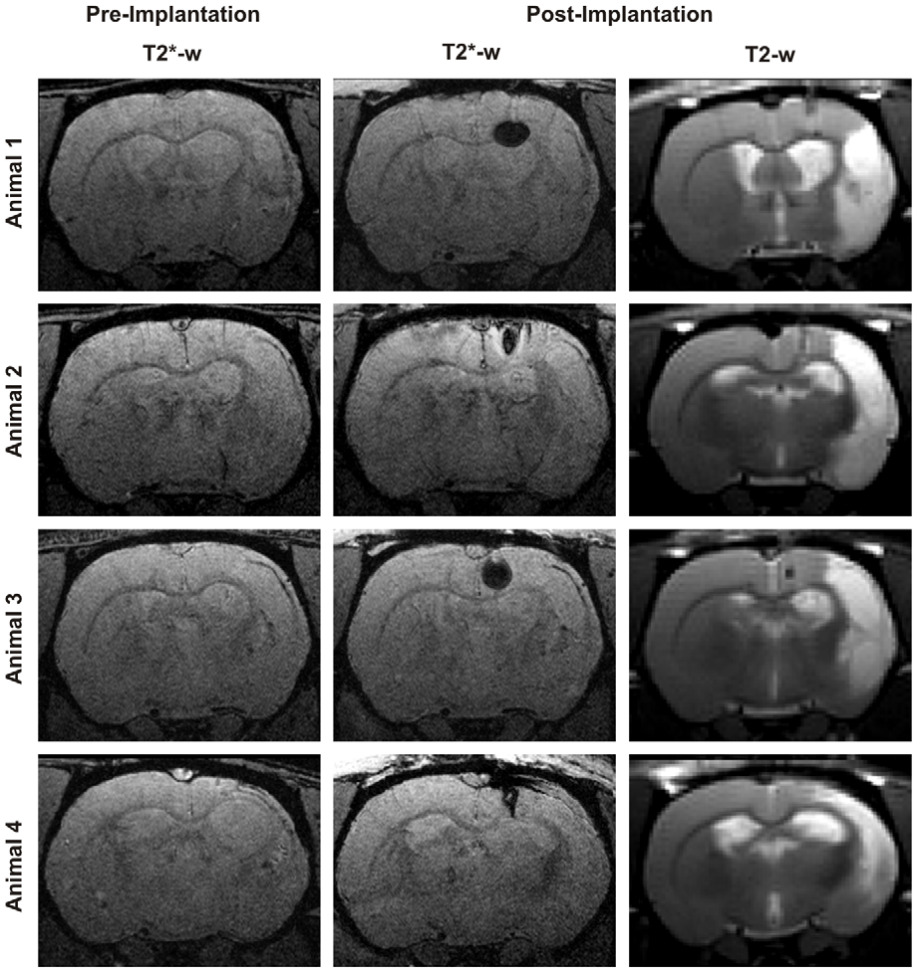
Localization of labeled cells by T2*-weighted MRI. 3D high resolution T2*-weighted MR images, pre- (1st column) and post- (2nd column) implantation of USPIO-labeled stem cells, showing the successful implantation of the cells into the brain parenchyma. Corresponding T2-weighted images (3^rd^ column) confirm that cells were implanted in healthy tissue.

Three weeks after occlusion of the MCA, and one day after the fMRI-based discrimination experiment, animals of group I (n = 4) were injected with 500.000 stem cells of the C17.2 line, with 10% of them labeled with iron oxide nanoparticles. Cell labeling was performed to ensure the accurate and successful positioning of the cells into the brain parenchyma, by acquiring T2*-weighted MR images directly following the implantation process (Fig. 3). Labeling was intentionally restricted to only 10% of all cells to exclude that the remaining 90% unlabeled cells could suffer alterations of their viability or cell characteristics, which could originate from the presence of the iron depots of the label in the cytoplasm. Cells were injected into healthy tissue, ipsilaterally and close to the lesion border, at a position caudal to bregma. The exact implantation coordinates varied among animals as they depended on the extension of the ischemic lesion of each individual, assessed on T2 maps obtained from T2-weighted MR images (Fig. 3).

### Bold Functional Imaging

**Figure 4:**
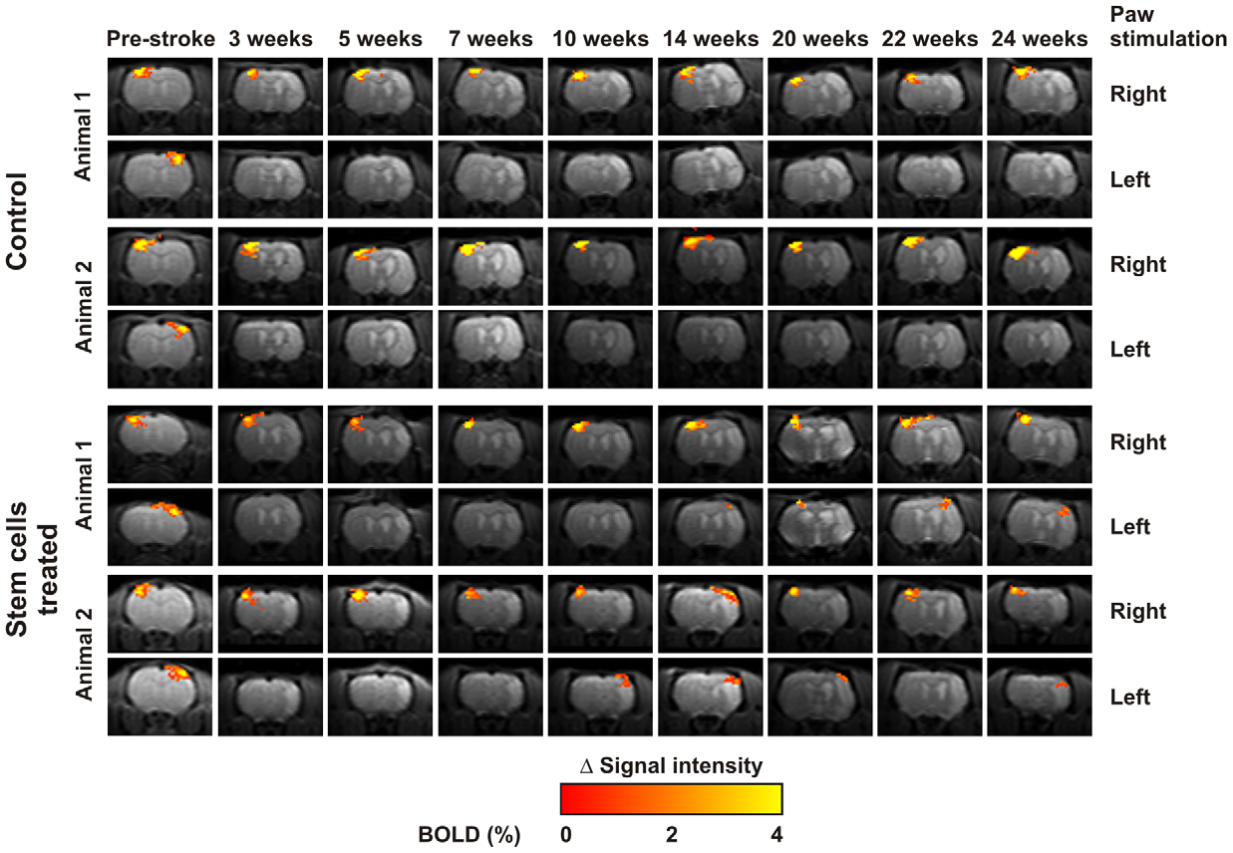
Functional brain recovery during six months observation. BOLD images obtained during the 24 weeks exploration period for two control animals (top rows), presenting a permanent loss of BOLD signal on the affected right hemisphere, and the two stem cell implanted animals (bottom rows) that regained BOLD activity at the affected hemisphere starting from week 10.

Both groups of animals (with and without stem cell implantation) were studied for a period of 6 months (24 weeks) after the ischemic insult, using a well-established non-invasive longitudinal fMRI protocol [10]–[11]. A temporal profile of the acquired BOLD activation in the somatosensory cortex is presented for 4 of the total 8 studied animals (2 implanted and 2 controls) in Fig. 4. None of the animals from the control group (II) recovered any sign of BOLD signals in the affected (right) hemisphere, while stable BOLD signals persisted throughout the whole study in the primary somatosensory representation field of the forepaw (S1_FL_) of the unaffected (left) hemisphere (Fig. 4, top rows). This result reaffirms the validity of the “sorting point” established at week 3, and previously reported results [10].

For the treated group (I), all animals showed a regular BOLD signal in the unaffected (left) hemisphere throughout the 6 months observation period. 2 of the 4 animals started to present signs of BOLD signals in the affected (left) hemisphere starting from weeks 10 to 14 after MCA occlusion (weeks 7–11 after implantation of cells). Activation was always observed at the original representation field of the forepaw in the sensorimotor cortex (S1_FL_), and no signs of plastic reorganization were observed on those brains (Fig. 4, lower rows). In one of these two animals, a transient BOLD signal was observed at week 20 in the healthy (left) hemisphere, when stimulating the left (ipsilateral) paw (Fig. 4, treated animal #2, week 20).

### Relation between Lesion Extent and Functional Recovery

**Figure 5:**
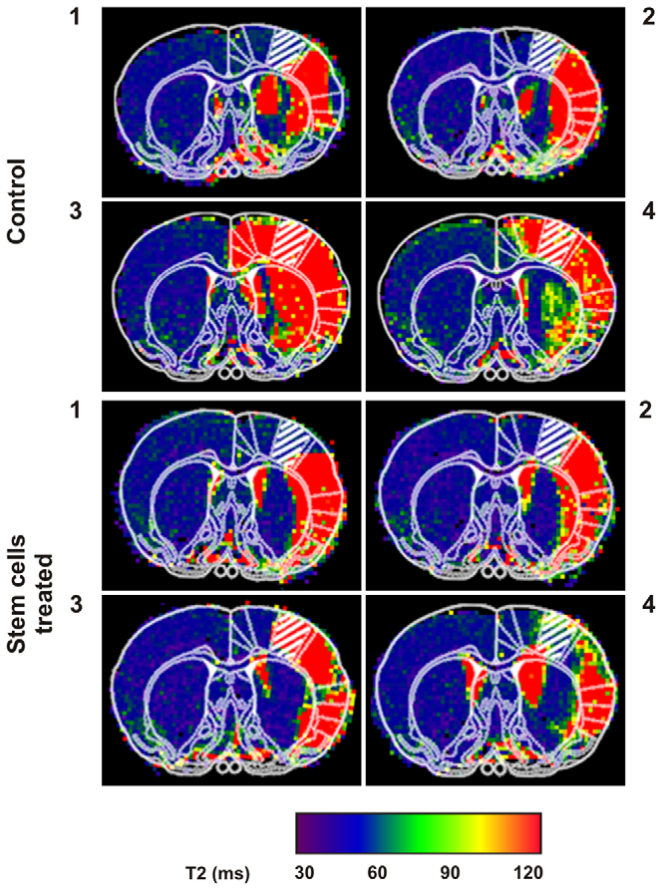
Spread of ischemic lesion. T2 maps of the 4 control (top) and 4 stem cell implanted (bottom) animals used in our study. The corresponding coronal sections of the Paxinos and Watson atlas of the rat brain [12], with the key brain region S1_FL_ shadowed, have been superimposed in each image, showing that animals labeled as 3 and 4 for each group presented lesions that include this region of the cortex, while in the others (animals 1 and 2 of each group) the S1_FL_ region is not affected.

In Fig. 5, T2 maps are presented documenting the extent of the lesion for all 8 studied animals (groups I and II). The MR images were superimposed with a map of the different anatomical regions of that section of the brain, taken from the anatomical atlas for the rat brain by Paxinos and Watson [12]. In those images, the S1_FL_ area ipsilateral to the affected hemisphere, was spared from the ischemic lesion for the two treated animals that recovered BOLD activity (animals 1 and 2 of group I), while the S1_FL_ area was actually part of the damaged tissue area in the case of the two animals not recovering activity (animals 3 and 4 of the treated group I in Fig. 5). Therefore, these latter 2 implanted animals had no chance to recover function, since the original representation field of the forepaw was destroyed by the ischemic lesion.

**Figure 6:**
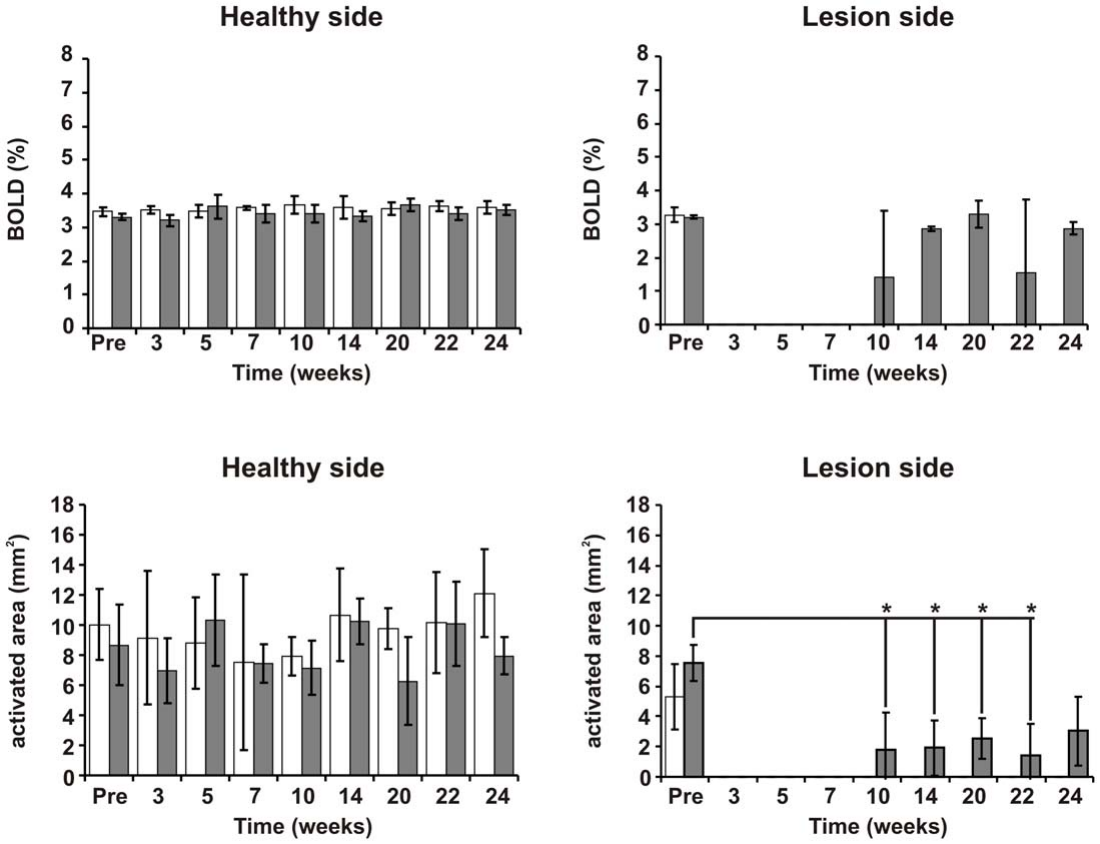
Quantitative analysis of fMRI data during recovery. *Top row*, percent change of signal intensity (BOLD effect) during stimulation of the forepaw (mean value ± standard deviation) for the healthy (left plot) and affected (right plot) hemispheres of the brain, for the control group (white bars), and for the two animals treated with stem cells that regained BOLD signal in the affected hemisphere (dark gray bars). *Bottom row*, activated volume (in mm^3^) in the primary somatosensory cortex in the healthy (left) and affected (right) hemispheres.

BOLD signals were quantified to obtain both, the percentage of signal intensity change during stimulation, and the extent of the activated brain volume in cubic millimeters. Results are presented in Fig. 6. For the healthy (left) hemisphere, all 8 animals presented similar values for both, BOLD signal intensity (mean±SD across all fMRI sessions; 3.43±0.15% for the control group *vs* 3.56±0.07% for the treated group) and activated volumes (mean±SD across all fMRI sessions; 8.35±1.55 mm^3^ for the control group *vs* 9.57±1.42 mm^3^ for the treated group). Results were not significantly different at a level of p<0.01. Values for the affected (right) hemisphere in the fMRI session prior to MCA occlusion (3.27±0.21% and 5.28±2.16 mm^3^ for control group II *vs* 3.20±0.05% and 7.52±1.20 mm^3^ for the treated group I) were not significantly different from the results of the left hemisphere.

On the affected (right) hemisphere, no activity was detected on BOLD images for any animal of the control group, at any time-point of the 6 months observation period, as well as for those two animals implanted with stem cells but with the ischemic lesion directly affecting the S1_FL_ area of the brain.

The two animals of the treated group, with the original representation field of the forepaw spared from the ischemic lesion, showed a BOLD response starting from week 10 after lesion induction (week 7 after implantation) for one of them and from week 14 (week 10 post implantation) for the other one. The mean value of the BOLD signal intensity for the affected (right) hemisphere over this period (weeks 10–24) was 2.4±0.86%, which is not significantly different from the values obtained for the healthy hemisphere, and from the pre-stroke scans on both brain hemispheres (cf. above). But activated representation volumes continuously increased during the observation period from 1.76±2.49 mm^3^ (week 10) to 3.04±2.16 mm^3^ (week 24). These values were significantly different, except for week 24, from pre-stroke values, or from values of the healthy (left) hemisphere for all time points, at a significance level of p<0.05.

Thus, treated animals which preserved an intact appearing original representation field of the forepaw, regained brain activity in that area, starting 7–11 weeks after implantation of stem cells, with the same intensity as prior to the lesion, but starting recovery in a significantly smaller volume which then continuously grew over time, without having (yet) reached normal values at the end of the 6 month observation period.

### Electrophysiology

**Figure 7:**
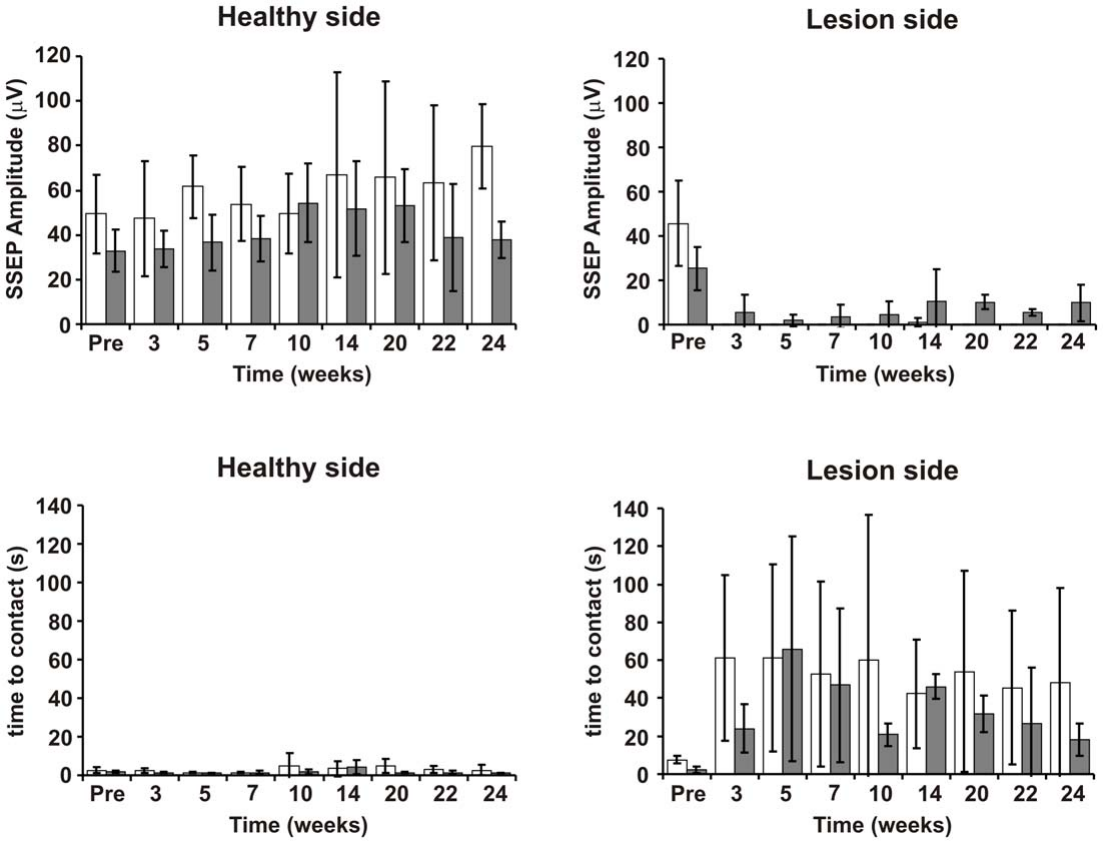
Quantitative analysis of electrophysiologic data during recovery. *Top row*, Amplitude (in µV) of the somatosensory evoked potentials detected over the S1_FL_ cortical area of the brain, acquired during the fMRI sessions. *Bottom row*, result of the sticky tape behavioral test. Time to contact the tape stuck to the forepaws of the animals is presented (mean ± standard deviation, in seconds). As in Fig. 4, the plots present the data for the control group (white bars), and for the group of animals treated with stem cells that recovered BOLD signal (dark gray bars).

During all fMRI sessions, somatosensory evoked potentials were recorded over the S1_FL_ areas of both forepaws with subcutaneously implanted electrodes over the intact skull of the rats (Fig. 7). No significant differences were observed for the amplitudes of SSEP signals recorded over the healthy (left) hemisphere of all 8 animals in all fMRI sessions (42.0±8.6 µV for treated group *vs* 59.7±10.6 µV for the control group), or the affected (right) hemisphere in the pre-stroke scan (26.2±6.7 µV for treated group *vs* 45.7±19.1 µV for the control group). For the untreated group, as well as for the two treated animals that had presented with an ischemic S1_FL_, no SSEP signals were detected at any of the sessions after MCA occlusion, confirming the absence of BOLD signal during those sessions. On the other hand, small but significant SSEP signals (3.64±5.14 µV) were detectable 4 weeks after implantation of stem cells (week 7 after MCAO). This return of electrical activity thus appeared 3 weeks earlier than the first time a BOLD response was achieved in the lesioned brain hemisphere. Those values increased continuously until week 14, when a value of around 10 µV (half of the value at the pre-stroke scans) was achieved. Then, this amplitude did not increase further but persisted for the following sessions (mean±SD of 8.98±2.3 µV for sessions at weeks 14, 20, 22 and 24).

### Behavioral Testing

Behavioral tests of adhesive tape removal from the paw were performed for all animals always the day before the fMRI session, and were preceded by 3 training sessions before animals were submitted to MCA occlusion. Results are presented in Fig. 7.

At base-line level, animals contacted the tape at very short latency times for both, the right paw (control group: 2.5±1.3 s *vs* treated group: 1.8±0.63 s) and the left paw (control group: 7.6±1.8 s *vs* treated group: 3.5±1.6 s). Results were not significantly different at a level of p<0.01.

For the unaffected (right) limb, controlled by the healthy (left) side of the brain, contact times remained at the same level for both groups during the whole 6 months observation period (control group: 2.9±1.2 s *vs* treated group: 1.7±0.9 s).

For the left forepaw, controlled by the affected (right) hemisphere of the brain, times to contact the tape were dramatically increased and ranged between 61.0±43.6 s at week 3 after stroke to 47.9±50.6 s at week 24, with a mean value of 53.1±45.0 s over the observation period of 6 months. The two animals that received stem cells, with ischemic S1_FL_ area, also presented large behavioral deficits, and the corresponding contact times (ranging from 60.2±8.7 s at week 3 to 24.5±14.8 s at week 24, with a mean value of 42.1±36.3 s) were not significantly different from those obtained for the (untreated) control group. For the two animals that received stem cells, and where the S1_FL_ area was not directly affected by the ischemic lesion, latency times ranged from 24.0±12.7 s at week 3 to 18.2±8.7 s at week 24, with a mean value of 35.0±26.1 s. We would like to point out the surprisingly low value at week 3 for these animals (test performed the day before stem cell implantation), which does not match with the much higher values obtained for the same animals at week 5 (65.8±59.2 s) or week 7 (46.9±40.4 s). This result is most likely due to the fact that, at this phase of the study, the number of animals per group is limited, and differences on the performance of one single animal at a particular session have large influence on the mean values, as stated by the high standard deviations of each mean. Although the mean value of the treated group did not reach a significant difference relative to the control group at any time point (at a level of significance of p<0.01), a trend toward a better behavioral performance of the treated animals, particularly from week 14 on to the end of the observation period, is clearly visible in Fig. 7.

### Histology

At the end of the study, 21 weeks after stem cell implantation, no trace of the implanted, β-galactosidase positive cells was observed on histological sections, and only traces of the iron load of the 10% labeled cells was found at the injection site (data not shown). Substantial integration of grafted stem cells into the periphery of the ischemic tissue was definitely not observed.

## Discussion

Experimental studies using stem cell implantation to treat stroke have mostly focused on the hypothesis of cell replacement mechanisms, i.e. the repair of damaged tissue for the recovery of lost functions. In vivo cell tracking experiments were designed to follow the fate of such cells after implantation, demonstrating that cells are able to survive, migrate, differentiate and integrate into existing neuronal networks [13]–[19]. Lately, however, it has been accepted that implanted cells do not have to integrate into existing neuronal networks, or repair damaged networks, but that their presence can already induce endogenous mechanisms of tissue repair and brain plasticity, by means of a paracrine activity like, for example, releasing trophic factors [5]–[6], [20]–[22]. The importance of these two different mechanisms for functional recovery after stroke mediated by the therapeutic intervention of stem cells is not completely known.

The effectiveness of stem cells to enhance functional recovery after stroke has been regularly reported during the last decade in the literature [4], [7]–[8], [23]–[31]. However, the positive results of these investigations were based on behavioral tests to assess the extent of regained functional activity, while the studies do not provide insight into the physiological mechanisms underlying the observations of outcome improvement.

It was the goal of the present study to elucidate the mechanism dominantly responsible for the observed functional outcome. For this purpose, we performed, for the first time, a longitudinal follow-up investigation on stem cell-mediated functional recovery after stroke by using functional neuroimaging tools, reinforced by the use of behavioral testing and measurements of electrical brain activity.

The major findings of our present study were: 1) Untreated, ischemic rats, included according to our rigorous selection criteria (cf above), did not demonstrate any spontaneous recovery process, neither by electrical activity criteria of SSEPs, neither by fMRI nor by behavioral standards. 2) Grafted stem cells did not integrate into the ischemic periphery to form a new neuronal network but were lost after 6 months despite persistent functional improvement. 3) Functional improvement is causally related to the grafting of stem cells. Return of electrical activity and fMRI signal in response to the forepaw stimulation was observed exclusively in stem cell treated animals. This return of activation in the original representation field was accompanied by an ongoing behavioral improvement pattern. 4) Stem cell mediated functional improvement was achieved exclusively through recovery of the original representation field. 5) There was no plastic reorganization either to an adjacent cortical area or even to the contralateral hemisphere of all animals studied, stem cell treated and untreated. Only in a single case and at one time point, we observed a shift of the activation field to the contralateral hemisphere, but this had shifted back already during the next study time point.

In sum, these observations lead us to conclude that the dominant reason for the observed return of functional activation several weeks after stem cell implantation must lie in the cells' contribution of a paracrine effect to the brain's own efforts for restitution of lost functions. This is in full agreement with several earlier reports of stem cell mediated outcome improvement where the authors interpreted their results as predominantly due to paracrine effects [5]–[6], [20]–[22].

### Animal Inclusion Criteria

After a thorough process of “animal screening”, based on anatomical and BOLD-fMRI data, we were able to distinguish and sort out those animals that would *spontaneously* regain functional activity in the affected brain hemisphere. Thus, all animals that passed through our “screening filter” did not spontaneously regain BOLD signal for a period of 6 months. This selection drastically reduced the number of animals in our study to a small fraction of the initial pool of animals (8 out of 51), but in return provided us with a rigorous protocol guaranteeing highly reliable data of the mechanisms behind the therapeutic effect of stem cells in the brain, while safely excluding naturally occurring, but confounding functional restoration processes.

Paradoxically, the extremely rigorous inclusion criteria used in this study constitutes one of its greatest strengths and, at the same time, its biggest limitation. From the initial 51 animals submitted to surgery, only 8 remained though the whole study, because we succeeded in separating confounding effects of spontaneous and stem cell-mediated processes of functional recovery after stroke. To our knowledge, there are no reports in the literature that have addressed this issue, while our data and conclusions are free of interferences caused by naturally occurring processes. This is a major strength of the present investigation. On the other hand, the validity of our conclusions may be hampered by the fact that only two groups of four animals each remained at the end of the study. The low number of animals per group in this study must be seen as a clear limitation and further work is warranted to confirm our results in larger groups of animals.

It should, however, also be emphasized in support of the study that the provision of the whole time profile for each individual during the longitudinal study is a potent aspect as it reduces the need for group averages of separate cohorts at each different study time point. Considering that in this investigation post-stroke fMRI alone has been performed at eight time points on eight animals, this would be equivalent to 64 animals needed for eight cohorts during invasive, one-time-point studies. Furthermore, the time profiles provide a quality of individual response characteristics otherwise not accessible with invasive studies of cohort comparisons.

### Time Line of Functional Recovery

Our “spontaneous-recovery-free” data clearly emphasize that the functional improvement of animals treated with stem cells after stroke does not take place on short term, but takes time before the therapeutic effect becomes detectable. This finding is in agreement with previously published works that depict a delayed action of implanted stem cells [32]. During the first 4 weeks after implantation no signs of recovery were observed. Then, animals started to show a progressive improvement that included re-appearance of electrical activity on the affected somatosensory cortex (weeks 4 to 7 post implantation), regaining of BOLD signal (weeks 7 to 10 post implantation) funneling into a robust response to stimulation in fMRI studies (21 weeks post implantation), but without yet reaching normal levels of activity at the end of the 6 month observation period.

A strong point of this study is that behavioral tests performed to assess functional outcome, electrical activity reflected in measured evoked potentials, and hemodynamic data from BOLD-based fMRI studies, were all well correlated and in agreement with previously reported data. In general, all point to the fact that some level of electrical activity starts to show up a few weeks before BOLD signal is regained, while behavioral tests do not completely reflect those effects, since compensatory mechanisms can induce a better-than-expected performance of the animals [10].

### Mechanisms of Functional Improvement

#### Reorganization versus recovery of original representation field

From a mechanistic point of view, our data confirm that, for this particular experimental model of stroke, stem cell mediation of functional recovery from stroke (as detected by SSEP and BOLD fMRI data) is associated with return of activity to the original representation field of the affected limb in the somatosensory cortex, while no plastic reorganization of the brain was observed in any of the 8 studied animals for the whole observation period of 6 months (except for a transitory effect for one single animal at a particular time point, already mentioned above). Our findings are in full agreement with literature, since we were not able to find a single report that supported the idea of stem cells inducing plastic reorganization after stroke. Furthermore, the present finding is extending the earlier report that already spontaneous recovery in this stroke model is exclusively taking place by reactivating the original representation field in the S1 cortex [10].

#### Cell replacement versus paracrine effects

We were not able to find implanted cells on histological sections (we only detected iron from that labeled 10% fraction of the implanted cells) at 6 months after their implantation, while at the same time monitoring a robust and continuously increasing functional improvement. There was definitely no neuronal network formation by the grafts observed. This lead us to speculate that the stem cells must have mediated functional recovery via a paracrine activity after implantation. Potential aspects could be release of growth factors, modulation of the immune response and enhancement of endogenous neurogenesis, rather than direct integration of the grafted cells into existing neuronal networks and their participation in processes of tissue repair. Although a better understanding is needed about the specific mechanism of such a paracrine effect, the present conclusions are in full agreement with earlier reports by several other laboratories, also showing paracrine effects [5]–[6], [20]–[22] as the most likely reason for observed behavioral outcome improvements. Survival of implanted stem cells, as was argued in those earlier reports, was not a prerequisite for efficacy, since the stem cells may be involved in paracrine activity early after implantation, and disappear after longer periods of time [22], [32]–[33].

A potentially occurring immune response to the cells after cessation of the immune suppression treatment with cyclosporin, must also be considered responsible for the absence of any trace of the implanted cells (of murine origin) at the end of the study. The loss of a considerable fraction of cells with time after suppression of immunologic treatment has already been addressed by other authors [33], but the fact that we continued immune suppression for a period of three weeks after stem cell implantation, appears to possibly have been a sufficient time period to allow paracrine effects to take place in therapeutically relevant and detectable extent. Nevertheless, we can not exclude that a more extended period for the immune suppressing treatment could have generated further therapeutic effects of the cells.

Finally, it should be mentioned that the here reported findings may be, to some extent, dependent on the used experimental stroke model and on the cell lineage chosen for treatment. While the intraluminal occlusion of the middle cerebral artery in the rat is one of the most used and validated experimental models of stroke with high clinical relevance, the selection of the C17.2 lineage of cells, a clonal multipotent neural precursor cell line originally derived from the external germinal layer of neonatal mouse cerebellum [34]–[35] was based on their reported good therapeutic effects in animal models of brain damage including stroke [36]–[38]. In a recent report of a regeneration model of spinal cord injury, the same C17.2 murine neural progenitor cell line as used in our study, was grafted into the lesion [39]. Those authors described a strong secretion by the C17.2 cells of the growth factors NGF, BDNF, and GDNF as the reason for their observation of axonal sprouting of endogenous cells and of the clear outcome improvement [39]. This report of a therapeutically relevant paracrine effect of the C17.2 cells is in full support of our present finding that these cells have not integrated but have, nevertheless, induced an unambiguous functional improvement.

Further studies will be required to investigate to what extent other cells can possibly participate in one or both of the discussed mechanisms after implantation (i.e. tissue repair vs. paracrine activity), depending on different experimental conditions.

### Conclusions

The careful exclusion of spontaneous improvement in our experimental protocol has provided unequivocal demonstration of stem cell-mediated functional improvement of the rat brain after stroke, as a consequence of stem cell grafting. The dominant mechanism of functional recovery is restitution of the activation of the original representation, without pronounced, detectable contributions from reorganizational processes. This functional restitution appears to be achieved primarily through paracrine activities of the grafted cells which have disappeared after several months despite persisting functional improvement. Due to the rigorous selection principle, the results have become extremely stable and reliable. But the resulting small sample number warrants further such complex longitudinal studies to confirm the present conclusions.

## Materials and Methods

All animal experiments were performed in accordance with the European Union's animal protection guidelines, and were approved by local authorities (Landesamt für Natur, Umwelt und Verbraucherschutz Nordrhein-Westfalen) under permission number 9.93.2.10.31.07.048 (dated 22.05.2007).

### Animals

51 male Wistar rats (Harlan-Winkelmann, Borchen, Germany) were used, with a weight of 308±23 g at the beginning of the study. Animals were kept on a 12/12 hours light/darkness cycle, with access to food and water *ad libitum*.

### Ischemic stroke model

The ischemic lesion was induced by transient (60 minutes) occlusion of the right middle cerebral artery (MCAO), using the intraluminal thread occlusion method, described elsewhere [10]. The occlusion period and the successful reperfusion of the right MCA were controlled by continuous recordings of ipsilateral laser-Doppler flowmetry (LDF).

### Anesthesia

During all surgical procedures, anesthesia was achieved with halothane (1.5–2%) in a gas mixture of O_2_:N_2_O (35%:65%). For fMRI studies, a medetomidine-based anesthesia protocol was used as described before [10]–[11].

### Stem cells

C17.2 cells, a clonal multipotent neural precursor cell line originally derived from the external germinal layer of neonatal mouse cerebellum [34]–[35], were cultivated in DMEM containing 15% FCS, non-essential amino acids (stock solution 1∶100), penicillin-streptomycin (stock solution 1∶100), mercaptoethanol, and leukemia inhibitory factor (100 nM). 10% of the cells were labeled with the iron-based MRI contrast agent SINEREM (generous gift of C. Corot, Guerbet, France), using a lipofecting reagent (FuGENE, Roche) [14]. 500.000 cells were injected into healthy tissue (as determined on T2-weighted MR images), ipsilateral to the lesion and at a position caudal to Bregma, depending on the axial extension of the ischemic lesion. The successful implantation of cells was confirmed by high resolution T2*-weighted MRI, with contrast created by the labeled 10% of the implanted cells. Cells were β-galactosidase positive, in order to be traceable in histological studies.

### Immunosuppression

To avoid an immune response to the implanted cells (from murine origin) in the rat, 25 mg/kg body weight of Cyclosporin A (Novartis) solved in castor oil (1 ml), was intradermally administered once every 2 days, starting on day 2 before stem cells implantation, and continued for 3 weeks.

### Immunohistochemistry

Animals were sacrificed after the last fMRI scan (week 24) under deep anesthesia and transcardially perfused with PBS and 4% paraformaldehyde (PFA). Brains were removed and postfixed overnight in 4% PFA, followed by immersion in a 30% sucrose solution for 3 days for cryo-protection, and stored at −80°C until further processing. Coronal 40 µm-thick sections were cut on a freezing microtome (Leica, Nussloch, Germany) and stored free-floating at 4°C. Prussian Blue iron staining was achieved by treating the tissue sections with 20% hydrochloric acid and 10% potassium ferrocyanide, trihydrate (Sigma-Aldrich). β-galactosidase staining of the Lac-Z positive injected stem cells was performed by treating the tissue sections with X-Gal, dissolved in DSMO, together with potassium ferrocyanide (Sigma-Aldrich). All tissue sections were analyzed and digitally photographed using Leica MZ FL III and Leica DM RB microscopes, both equipped with CCD cameras.

### Lesion follow-up

fMRI experiments were performed 1 week before and 3, 5, 7, 10, 14, 20, 22 and 24 weeks after stroke induction. Lesion anatomy was assessed using T2 maps generated from spin-echo images acquired on day 2 after MCA occlusion, and on all fMRI sessions. High resolution 3D T2*-weighted images were acquired on week 3, one scan before (control) and one after the injection of iron-loaded stem cells, and at 6, 8, 9, 13 and 24 weeks after MCAO. A schematic representation of the experimental protocol performed for each individual animal is presented in Fig. 1.

### MRI

Experiments were conducted on a 7T horizontal bore magnet (BioSpec, Bruker BioSpin, Ettlingen, Germany), equipped with actively shielded gradient coils (20 cm diameter, 200 mT/m). Radiofrequency transmission was performed with a home-built Helmholtz coil (12 cm diameter); signal was detected using a 2.2 cm circular surface coil, positioned over the head of the animal. The animal was fixed in a stereotaxic holder with teeth bar, earplugs and adhesive tape. Both, transmission and reception RF coils, were actively decoupled from each other.

### Lesion characterization

Lesion characterization, concerning size and status, was achieved using T2 maps which had been calculated from a set of multi-slice multi-echo (MSME) spin echo images: 16 echoes with TE/TR = 7.5/2000 ms, 16 slices of 1 mm thickness, FOV of 32×32×16 mm^^3^^ covered by a 128×128×16 matrix, resulting in an in-plane resolution of 250 µm.

### Cell implantation success

Cell implantation success was stated from high resolution 3D T2*-weighted images using a 3D-FLASH sequence, with a flip angle of 30 degrees, TE = 20 ms, TR = 200 ms, a FOV of 20×20×10 mm^3^ covered by a 160×160×40 points matrix, zero-filled to 256×256×64 points to give a nominal resolution of 78×78×156 µm.

### Functional MRI

Functional MRI experiments were performed as described earlier [10]–[11], acquiring sets of 115 consecutive spin-echo Echo-Planar-Images (SE-EPI; TE/TR = 30/3000 ms, 5 slices of 2 mm thickness, FOV of 25.6×25.6 mm^2^ covered by a 64×64 matrix, i.e. an in-plane resolution of 400 µm). With this protocol, the total acquisition time was 445 seconds, segmented in a fMRI paradigm of 6 resting-state periods of 45 seconds (15 images each) and 5 intercalated stimulation-state periods of 15 seconds (5 images each). This paradigm was repeated typically 5 times for each forepaw, allowing a resting period of at least 10 minutes between runs.

### BOLD

BOLD functional activation imaging was achieved by electrical stimulation of both forepaws in an alternate fashion, using subcutaneous needle electrodes and a DC stimulation current at intensity (2 mA, pulse length 0.3 ms; 3 Hz) below pain threshold (STG1004 stimulator from Multi Channel Systems, Germany).

### Image processing

All MRI images were processed using self-developed applications for the Image-J software platform [40]–[41]. For fMRI studies, statistical parametric activation maps were constructed with the software STIMULATE [42]. The time course of each pixel during forepaw stimulation was examined using a paired Student's t-test (p<0.05). Only clusters that included at least four adjacent activated pixels were considered as activated areas [43].

### Electrophysiology

During the fMRI sessions, short latency somatosensory evoked potentials (SSEPs) were recorded bilaterally from the primary somatosensory cortices of the intact skull (±3.5 mm lateral, 0 mm rostro-caudal from bregma), using subcutaneously inserted steel needle electrodes. SSEPs were constructed with DasyLab (DATALOG, Mönchengladbach, Germany) by averaging the signal recorded from the brain during stimulation of the paw with 100 triggered rectangular electrical pulses (2 mA;3 Hz;0.3 ms). Recorded signal was amplified 1000-fold and filtered with a bandpass filter between 5 and 1.000 Hz for displaying purposes.

### Behavioral testing

The adhesive tape removal test was used to evaluate sensorimotor deficits [44]–[45]. Two circular-shaped strips of tape (12 mm of diameter) were applied, in random order and with equal pressure, to the saphaneous part of the forepaws. Animals were observed while removing the tapes in their home cages. Latencies to contact and to remove the right and left tapes from the paws were recorded in three trials per session. The mean time of the three trials was calculated. At least five minutes of rest was allowed between each trial. Healthy animals were trained daily for 3 days before baseline session, which was performed the week before the first fMRI session (pre-scan in Fig. 1). After MCA occlusion, behavioral tests were performed always the day prior to the fMRI session.

### Statistical analysis

Statistical analysis was performed with SPSS V15.0. All values are expressed as mean ± SEM, unless stated otherwise. Because of sample sizes, statistical comparisons were performed using Kruskal-Wallis ANOVA.
